# Treatment of the Fracture of the Clavicle

**Published:** 1844-12

**Authors:** Jno. Evans

**Affiliations:** Attica, Ind.


*Treatment of the Fracture of the Clavicle.* By Jno. Evans, M. D., of Attica, Ind.

There is no fracture so generally maltreated, and yet so easily managed, as that of the clavicle. This may be attributed to the complicated and bungling apparatus of Dessault; which, notwithstanding its inefficiency in meeting the indications of cure, from the recommendation of authors, is generally resorted to by the profession.* In ten cases where there had been fracture of the clavicle submitted to my examination, I did not find one in which the fractured extremities had been in coaptation at the time of reunion; and upon inquiry I found that the dressing of Dessault had been, in each case, more or less perfectly applied and relied upon.

The deformity produced by a fracture of the clavicle, is a drooping of the injured shoulder, with a slight approximation to the chest.

The only treatment necessary is to bring the fractured ends of the bone into coaptation, and to keep them there. As taught by the advocates of the figure 8 bandage, the proper elevation of the shoulder places the clavicle in its natural position. As the approximation of the shoulder to the chest depends upon its depression, and the consequent misplacement of the fractured extremities of the bone, its elevation brings those extremities into coaptation, which causes the bone to assume a position, in which by resistance lengthwise, it counteracts the tendency to approximate the chest. But for the difficulty in preventing pressure upon the clavicle itself, the figure 8 bandage would be all that is necessary; as drawing the shoulders directly backward, will, by the counteraction of the rhomboid muscles, elevate them. In one case I succeeded so well, that it is difficult to distinguish the fractured bone from the sound one: by the application of a pillow compressed and rolled into a pad, between the shoulders, upon which a straight stick was placed, extending outward on each side as far as the point of the acromion, forming a neck-yoke, from each end of which a padded stirup passed under the arm, which was drawn sufficiently tight to elevate the shoulders. The only objection to this apparatus is its inconvenience.

I have recently adopted, with entire success, a dressing made by applying a linen or drilling sleeve, extending from the middle of the forearm to a few inches above the elbow, stitched on the arm so as to fit closely when flexed to a right angle, covering the point of the elbow, and crossing it in a direction to fit smoothly when applied: a strap of linen or muslin four or five inches wide, was firmly stitched to the under surface of the sleeves; the end of it next the body was carried across the breast, and the other passed over the elbow across the back; these ends were drawn together over the opposite shoulder, sufficiently tight to keep the bone in place, and fastened upon a pad. A band was passed around the wrist and stitched to the strap passing across the breast, to support and confine the forearm. This dressing meets every indication, in a simple fracture of the clavicle. It has the advantage of simplicity, permanency, and cheapness—can in a few minutes be made extemporaneously—while it is the most convenient and easily worn. The only inconvenience liable to occur, is in a deficiency of padding on the shoulder, allowing excoriation.

Attica, Ind., Dec. 1844.

